# Direct activation of tRNA methyltransferase-like 1 (Mettl1) gene by thyroid hormone receptor implicates a role in adult intestinal stem cell development and proliferation during *Xenopus tropicalis* metamorphosis

**DOI:** 10.1186/s13578-020-00423-1

**Published:** 2020-05-04

**Authors:** Wonho Na, Liezhen Fu, Nga Luu, Yun-Bo Shi

**Affiliations:** Section on Molecular Morphogenesis, National Institute of Child Health and Human Development, National Institutes of Health, Bethesda, MD 20892 USA

**Keywords:** Thyroid hormone receptor, *Xenopus tropicalis*, Anuran metamorphosis, Methyltransferase, tRNA, Stem cell, Intestine

## Abstract

**Background:**

Thyroid hormone (T3) plays an important role in vertebrate development. Compared to the postembryonic development of uterus-enclosed mammalian embryos, T3-dependent amphibian metamorphosis is advantageous for studying the function of T3 and T3 receptors (TRs) during vertebrate development. The effects of T3 on the metamorphosis of anurans such as *Xenopus tropicalis* is known to be mediated by TRs. Many putative TR target genes have been identified previously. Among them is the tRNA methyltransferase Mettl1.

**Results:**

We studied the regulation of Mettl1 gene by T3 during intestinal metamorphosis, a process involves near complete degeneration of the larval epithelial cells via apoptosis and de novo formation of adult epithelial stem cells and their subsequent proliferation and differentiation. We observed that Mettl1 was activated by T3 in the intestine during both natural and T3-induced metamorphosis and that its mRNA level peaks at the climax of intestinal remodeling. We further showed that Mettl1 promoter could be activated by liganded TR via a T3 response element upstream of the transcription start site in vivo. More importantly, we found that TR binding to the TRE region correlated with the increase in the level of H3K79 methylation, a transcription activation histone mark, and the recruitment of RNA polymerase II by T3 during metamorphosis.

**Conclusions:**

Our findings suggest that Mettl1 is activated by liganded TR directly at the transcriptional level via the TRE in the promoter region in the intestine during metamorphosis. Mettl1 in turn regulate target tRNAs to affect translation, thus facilitating stem cell formation and/or proliferation during intestinal remodeling.

## Introduction

Thyroid hormone (T3) plays important roles for organ development and also regulates homeostasis and physiological function of many adult organs/tissues in vertebrates [1–8]. Insufficient T3 during human development leads cretinism, which results in growth retardation, developmental delay, and impaired mental development [3–8]. During human development, the level of T3 in the plasma is gradually elevated from a few months prior to birth, and peaks around birth, and gradually decreases for several months after birth to a steady adult level. This period of changing T3 level is called postembryonic period, when many organs mature into their adult forms. The studies of T3 function during mammalian development have been difficult, in part because the uterus-enclosed mammalian embryos are not easily manipulatable models. Moreover, the analysis of in vivo T3 action in mammals is difficult due to the influence of maternal T3. On the other hand, anurans, such as *Xenopus laevis* and *Xenopus tropicalis*, are an ideal animal model for studying how T3 affects postembryonic development, largely because they develop externally and are independent of maternal influence [3, 4].

During postembryonic development, the tadpole undergoes metamorphosis to become a frog in a process that is totally controlled by T3 and can be easily manipulated via the availability of T3 to the rearing tadpoles [3, 4]. Anuran metamorphosis involves drastic changes in essentially every organ/tissue of a tadpole, including de novo development of adult organs, complete resorption of larval ones, and remodeling of others. Many processes during metamorphosis are similar to those during mammalian postembryonic development, including the maturation and the remodeling of the brain, intestine, and the lung, etc. Such properties make anuran metamorphosis a unique model to study how T3 regulates postembryonic development.

Numerous studies have shown that T3 affects metamorphosis by regulating gene transcription through T3 receptors (TRs) [9–35]. TRs are DNA-binding transcription factors that form heterodimer with 9-cis retinoic acid receptors (RXRs) [1, 2, 4, 14, 36–39]. These heterodimers bind to T3 response elements (TREs) in the promoters of T3-inducible genes, and activate or repress the transcription of target genes by recruiting cofactors in a T3-dependent manner. In order to identify direct TR target genes during metamorphosis, we have previously performed a ChIP (chromatin immunoprecipitation)-on-chip analysis of TR binding in the intestine of premetamorphic tadpoles treated with or without T3 and identified about 300 genes bound by TR in the tadpole intestine [40]. Among these putative TR target genes is methyltransferase-like 1 (Mettl1), a methyltransferase for *N*^7^-methylguanosine (m^7^G) tRNA.

In eukaryotes, the posttranscriptional modifications of tRNAs, including methylation, can control tRNA folding, stability, and function in mRNA translation [41–43]. Dysregulation of tRNA modifications is related to genetic disorders and cancers [44, 45]. The modification of the m^7^G at position 46 (m^7^G46) of tRNAs is one of the most well-known tRNA modifications found in prokaryotes, eukaryotes and in some archaea [46]. In mammals, Mettl1 functions as a Wdr4 (WD-repeat domain 4)-containing methyltransferase for m^7^G tRNA [47, 48]. Knockout of Mettl1 in mESCs (mouse embryonic stem cells) reduces the stabilities of target tRNAs [47]. Furthermore, Mettl1 depletion causes ribosome pausing at m^7^G tRNA-dependent codons in mRNAs of cell cycle genes, and results in impaired mESCs self-renewal [47].

Intestinal remodeling involves near complete apoptotic degeneration of the larval epithelium and de novo formation of adult intestinal epithelial stem cells, followed by their proliferation and differentiation to form a complex, multiply folded adult intestinal epithelium [49–56]. Thus, it is likely that Mettl1 is induced by T3 to affect cell cycle progression during adult intestinal epithelium development.

Toward determining the role of Mettl1 during intestinal remodeling, we investigated the expression profiles of Mettl1 during natural and T3-induced metamorphosis and whether Mettl1 is regulated directly by TR at the transcription level. We discovered that Mettl1 expression peaks at the climax of intestinal remodeling when stem cells are forming and proliferating and that a TRE in the Mettl1 promoter region mediates the activation of the gene by liganded TR, which is accompanied by increased histone modification and the recruitment of RNA polymerase. Our findings suggest that Mettl1 is activated early during intestinal remodeling to facilitate adult intestinal stem cell development and/or proliferation.

## Results

### *Xenopus tropicalis* Mettl1 is upregulated in the intestine during natural and T3-induced metamorphosis

**Fig. 1:**
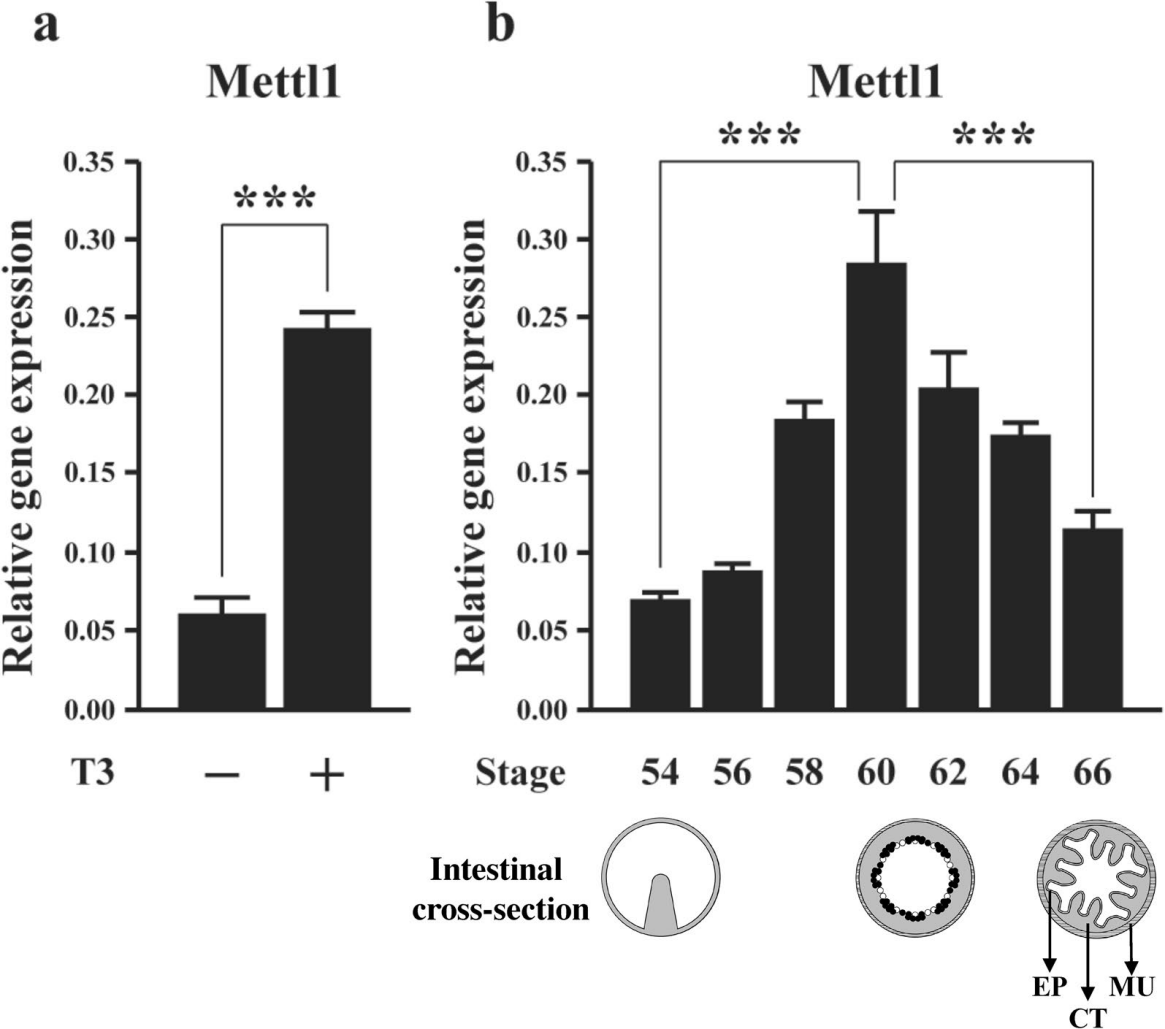
Expression of *Xenopus tropicalis* Mettl1 increases during T3-induced and natural metamorphosis. **a** The expression of Mettl1 was analyzed during T3-induced metamorphosis. Stage 54 tadpoles were treated with 10 nM T3 for 2 days and total RNA was isolated from the intestine for RT-PCR analysis. **b** During natural metamorphosis, Mettl1 expression gradually increased from premetamorphic period to peak at the metamorphic climax. Total RNA was isolated from the intestine of tadpoles at indicated stages for RT-PCR analysis. Shown below the expression data are schematic diagrams of the intestine at different stages. In premetamorphic tadpoles at stage 54, the intestine is a simple structure with a single epithelial fold, the typhlosole, and thin layers of connective tissue and muscles. At the metamorphic climax around stage 61, the larval epithelial cells begin to undergo apoptosis, as indicated by the open circles. Concurrently, the proliferating adult stem cells are formed de novo via dedifferentiation of some larval epithelial cells, as indicated by black dots. By the end of metamorphosis at stage 66, the newly developed adult epithelium (EP) has multiple folds, surrounded by thick layers of connective tissue (CT) and muscles (MU). L: intestinal lumen. All data represent mean ± S.E.M. Significance value was ***P ≤ 0.005

Because of the causative role of T3 on amphibian metamorphosis, the identification of Mettl1 as a putative target of TR from our earlier ChIP-on-chip analysis of the tadpole intestine [40] suggests that Mettl1 is regulated by T3 during intestinal remodeling. To investigate this possibility, we treated premetamorphic *Xenopus tropicalis* tadpoles at stage 54 with T3 for 2 days and analyzed Mettl1 expression in intestine by qRT-PCR. As shown in Fig. 1a, Mettl1 expression was increased dramatically in the tadpole intestine after T3 treatment, suggesting that T3 activates the gene directly via TR. In addition, when we analyzed the expression of Mettl1 in the intestine during natural metamorphosis, we found that its expression was low at the premetamorphic stage 54, increased gradually during metamorphosis to peak at metamorphic climax (stages 58–62), and subsequently dropped to a lower level at the end of metamorphosis (stage 66) (Fig. 1b). Thus, high levels of Mettl1 expression correlate with drastic remodeling of the intestine when larval epithelial cell death and adult epithelial stem cell formation/proliferation take place during stages 58–62 [49, 50, 57]. These findings suggest that Mettl1 is regulated by T3 and likely participates in intestinal remodeling during metamorphosis.

### *Xenopus tropicalis* Mettl1 encodes an evolutionally highly conserved methyltransferase and has a putative TRE in its promoter region

**Fig. 2:**
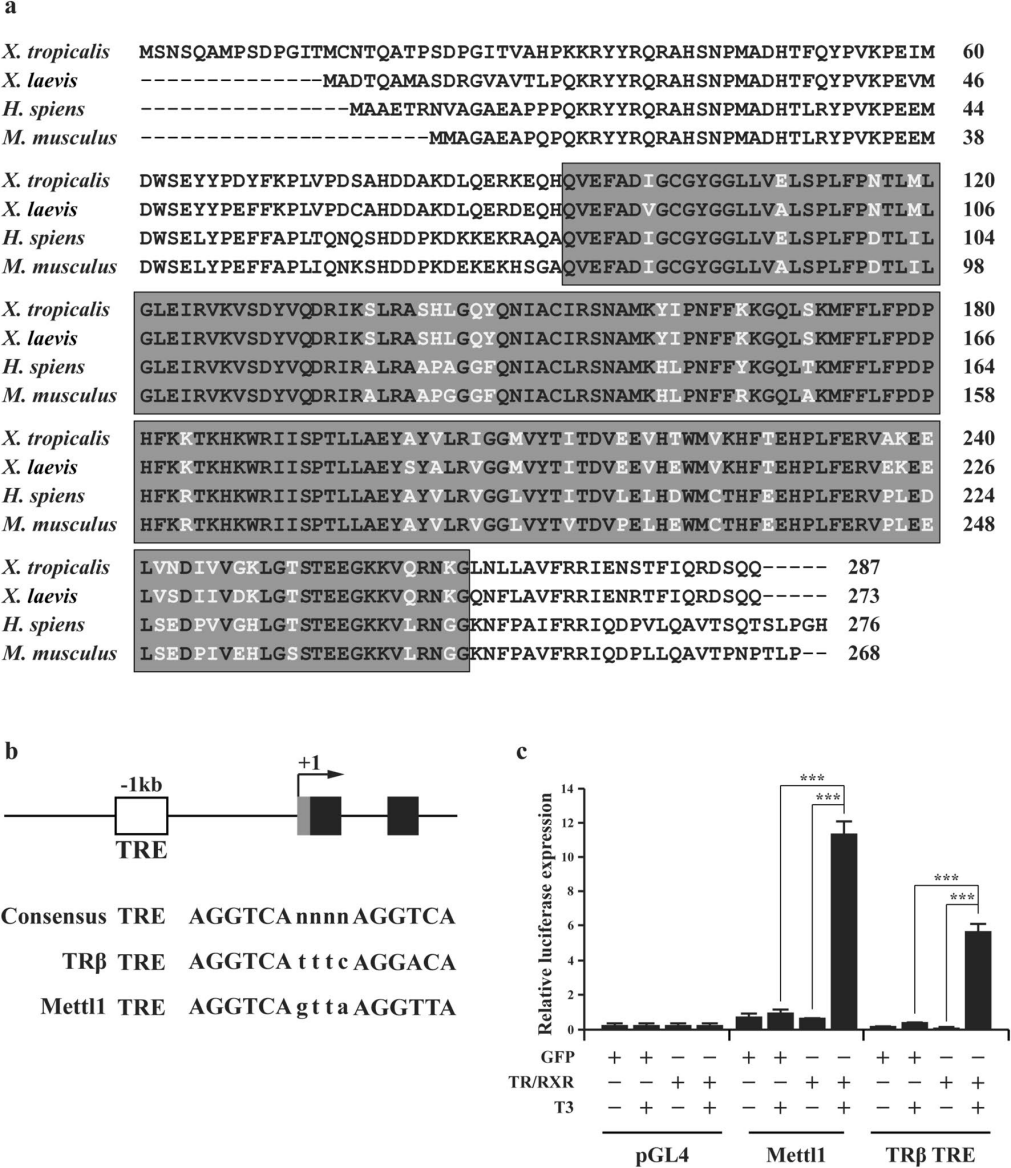
Mettl1 *methyltransf_4* domain is highly conserved evolutionally and liganded TR enhances *Xenopus tropicalis* Mettl1 promoter activity in vivo. **a** Amino acid alignment of Mettl1 from *X. tropicalis*, *X. laevis*, *H. sapiens* and *M. musculus*. The boxed region is the *methyltransf_4 domain*. Shaded amino acids indicate conserved residues. **b** Schematic representation of Mettl1 promoter and the first two exons. The putative TRE is shown as a white box. Gray box indicates the putative 5′ UTR of Mettl1 and black boxes indicate exons. The putative TRE is located at − 1128 bp from the predicted transcription start site of Mettl1 gene. **c** The Mettl1 promoter is activated by liganded TR in *Xenopus* oocytes. pGL4 was used as a negative control, and TRβ promoter construct was used as a positive control. The oocytes were injected with indicated mRNAs and reporter and harvested for luciferase assay. All data represent mean ± S.E.M. Significance value was ***P ≤ 0.005

The full-length coding region of *Xenopus tropicalis* (*X. tropicalis*) Mettl1 were obtained from GenBank. We identified a *methyltransf_4* domain in the predicted amino acid sequence by using Conserved Domains database. Comparison of the *methltransf_4* domain from *X. tropicalis*, *Xenopus laevis* (*X. laevis*), *Homo sapiens* (*H. sapiens*), and *Mus musculus* (*M. musculus*) revealed that *X. tropicalis* Mettl1 shares 95%, 83%, and 81% of amino acid sequence identity with *X. laevis*, *H. sapiens*, and *M. musculus*, respectively (Fig. 2a), suggesting that *Xenopus tropicalis* Mettl1 encodes a highly conserved protein that functions as a methyltransferase for m^7^G tRNA.

Sequence analysis of the predicted 5′ flanking region of the *Xenopus tropicalis* Mettl1 gene revealed the presence of a putative TRE located at -1128 bp from predicted transcription start site (Fig. 2b). The TRE sequence is very close to the consensus TRE composed of two direct repeats of AGGTCA separated by 4 nucleotides and similar to the well-characterized strong TRE of the *Xenopus laevis* TRβ gene [58] (Fig. 2b). To investigate if the putative TRE can mediate the transcriptional regulation of the Mettl1 gene by T3, we carried out luciferase reporter assay in *X. laevis* oocyte. In vitro synthesized mRNAs for TR and RXR or GFP were microinjected into *X. laevis* oocyte cytoplasm to allow the synthesis of the protein(s). Then, a luciferase reporter construct driven by the Mettl1 promoter was microinjected together with the control *Renilla* luciferase construct into the oocyte nuclei. After overnight incubation of the injected oocytes in presence or absence of T3, the oocytes were harvested for dual luciferase assay. The ratio of firefly luciferase to *Renilla* luciferase activities was then determined as the activity of the Mettl1 promoter. When TR/RXR overexpressed oocytes were treated with T3, the activity of Mettl1 promoter was strongly increased, compared to the non-treated oocytes or oocytes without TR/RXR overexpression (Fig. 2c). These findings were similar to those obtained with the luciferase reporter construct driven by the well-characterized, T3-inducible TRβ promoter used as positive control (Fig. 2c). Collectively, these findings indicate that Mettl1 promoter is activated by liganded TR/RXR.

### TR/RXR regulates the Mettl1 promoter via the putative TRE

**Fig. 3:**
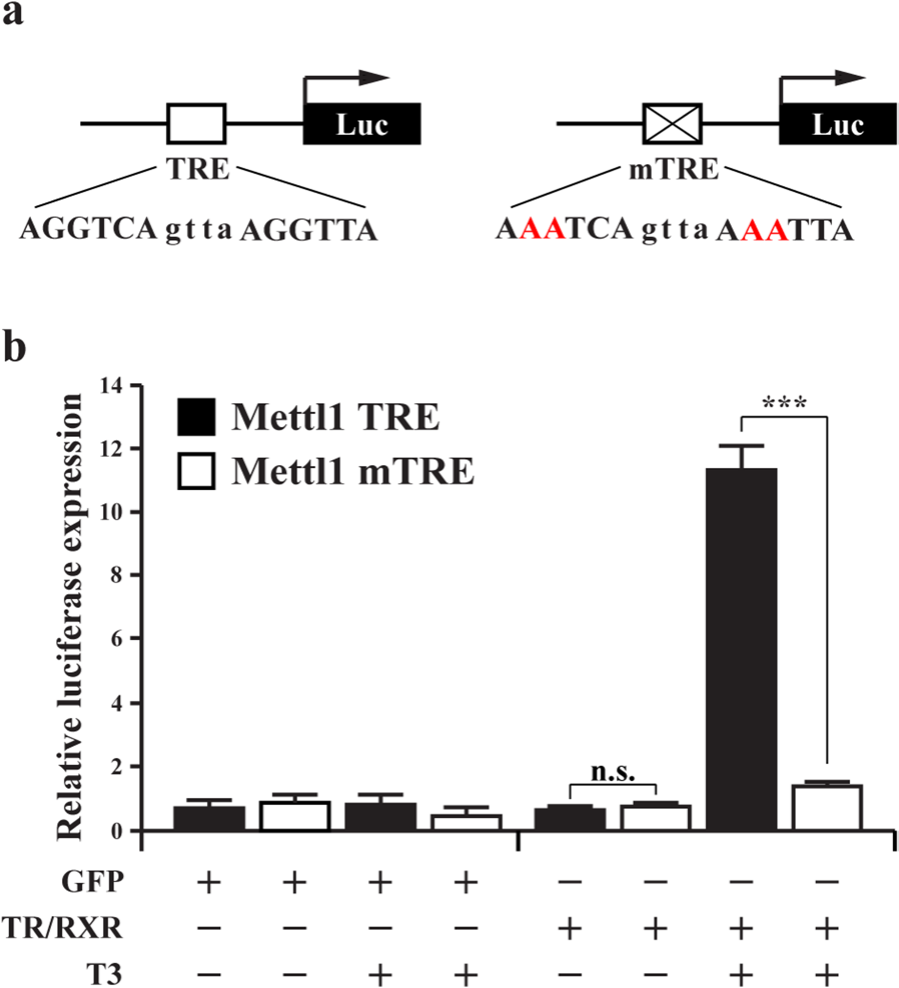
Liganded TR activates the Mettl1 promoter through the putative TRE. **a** Schematic diagrams of the Mettl1 promoter construct with the putative TRE of Mettl1 (Mettl1 TRE) or a mutant TRE (Mettl1 mTRE). The TRE sequence is shown below the TRE with the mutated residues shown in red. The Mettl1 TRE and Mettl1 mTRE fragments were cloned into pGL4.10 luciferase reporter vector. **b** The Mettl1 wild type but not the mutant promoter construct is activated by liganded TR in *Xenopus laevis* oocytes. The oocytes were injected with indicated mRNAs and reporter and harvested for luciferase assay. All data represent mean ± S.E.M. Significance value was ***P ≤ 0.005. *n.s.* not significant

To determine if the putative TRE is responsible for the activation of the promoter by T3 in the presence of TR/RXR, we mutated the putative TRE (Fig. 3a) and performed luciferase assay with wild type and mutated TRE promoter constructs in the oocytes as above. The results showed that when the putative TRE was mutated, the promoter could no longer be activated by liganded TR/RXR. These results indicate that putative TRE of Mettl1 promoter is a functional TRE, and liganded TR/RXR regulates the expression of Mettl1 gene through this TRE.

### TR binds to the putative TRE in premetamorphic tadpole intestine and increases histone H3K79 methylation and RNA polymerase II recruitment in response to T3 treatment

**Fig. 4:**
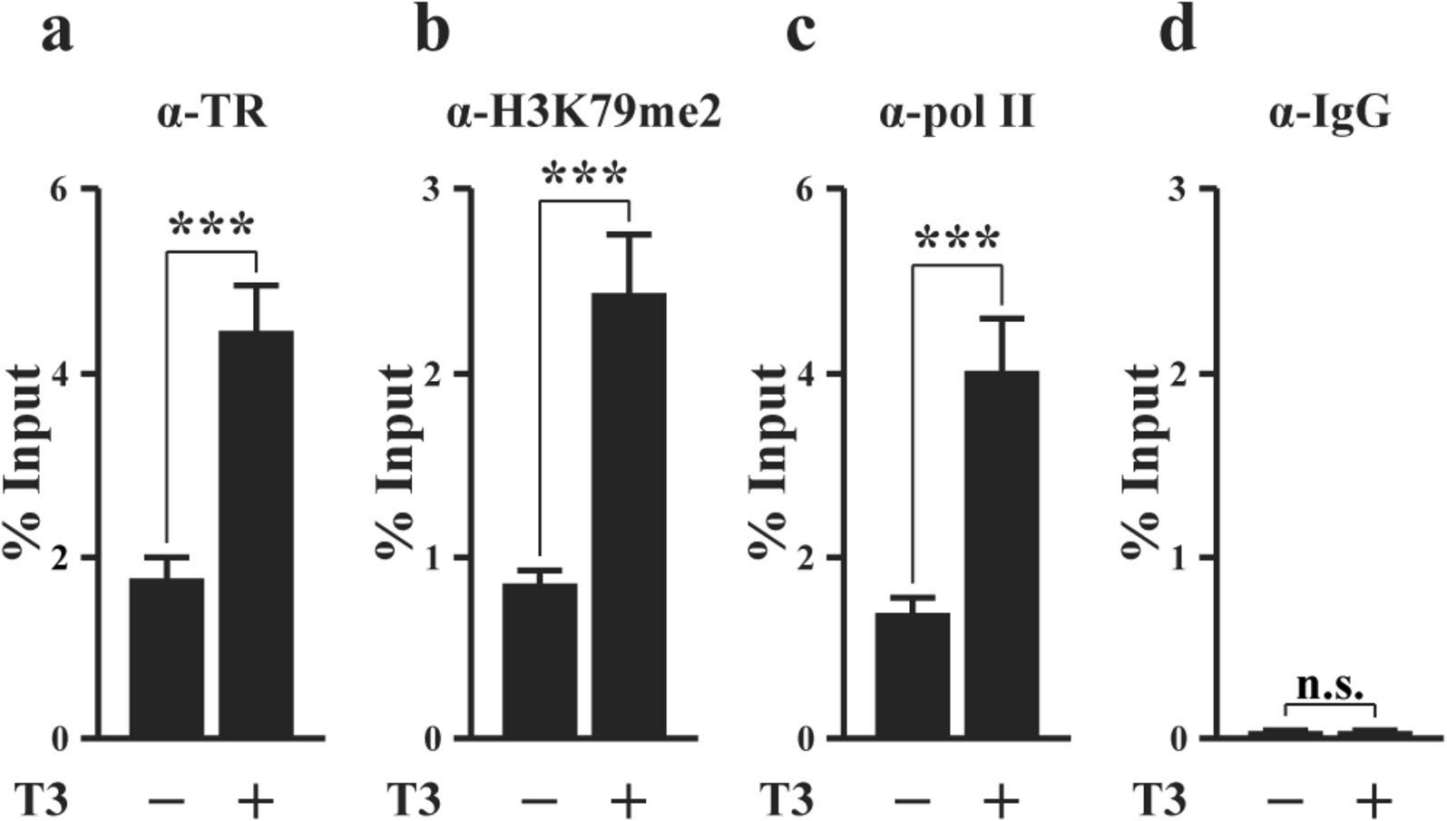
TR binds to the TRE in the Mettl1 promoter region in the intestine during T3-induced metamorphosis. ChIP assays were performed with indicated antibodies on the intestine of premetamorphic tadpoles treated with or with T3. **a** Liganded TR is present in the TRE region of Mettl1 promoter in the intestine of premetamorphic tadpoles and the binding is increased by T3 treatment. **b** The level of di-methylated H3K79, a histone mark for transcription activation, increases in the TRE region after T3 treatment. **c** RNA polymerase II is recruited to the Mettl1 TRE region after T3 treatment. **d** Only background ChIP signal is detected with the negative control IgG ChIP in the presence or absence of T3 treatment. All data represent mean ± S.E.M. Significance value was ***P ≤ 0.005. *n.s.* not significant

To determine if the putative TRE is functional in vivo during metamorphosis, we first analyze if TR is bound to the TRE in the intestine by ChIP assay on intestinal chromatin isolated from premetamorphic tadpoles at stage 54 treated with or without T3 for 2 days. The polyclonal anti-TR antibody (anti-TR) [59] was used for precipitation of TR-bound DNA and IgG was used as a negative control. The precipitated DNA was analyzed by using PCR primers for the TRE region. The results showed that TR was bound to the TRE region in premetamorphic tadpole intestine and T3 treatment enhanced the occupancy of TR at the TRE (Fig. 4a). To study if TR binding to the TRE is important for Mettl1 expression, we carried out ChIP assay with antibodies against RNA polymerase II and di-methylated histone H3K79 (H3K79me2), a known histone activation mark that is increased at TR target genes during metamorphosis [60, 61]. We observed that T3 treatment enhanced the recruitment of RNA polymerase II and the methylation of H3K79 in the Mettl1 TRE region (Fig. 4b, c). On the order hand, ChIP assay using normal IgG as a negative control showed the expected background signal both in the presence or absence of T3 (Fig. 4d). These results indicate that liganded TR is bound to Mettl1 TRE in tadpole intestine and enhances local histone modification and RNA polymerase II recruitment to activate the Mettl1 promoter during T3-induced metamorphosis.

### TR binding to Mettl1 TRE in tadpole intestine peaks at the metamorphic climax and correlates with peak levels of RNA polymerase II recruitment and histone H3K79 methylation during natural metamorphosis

**Fig. 5:**
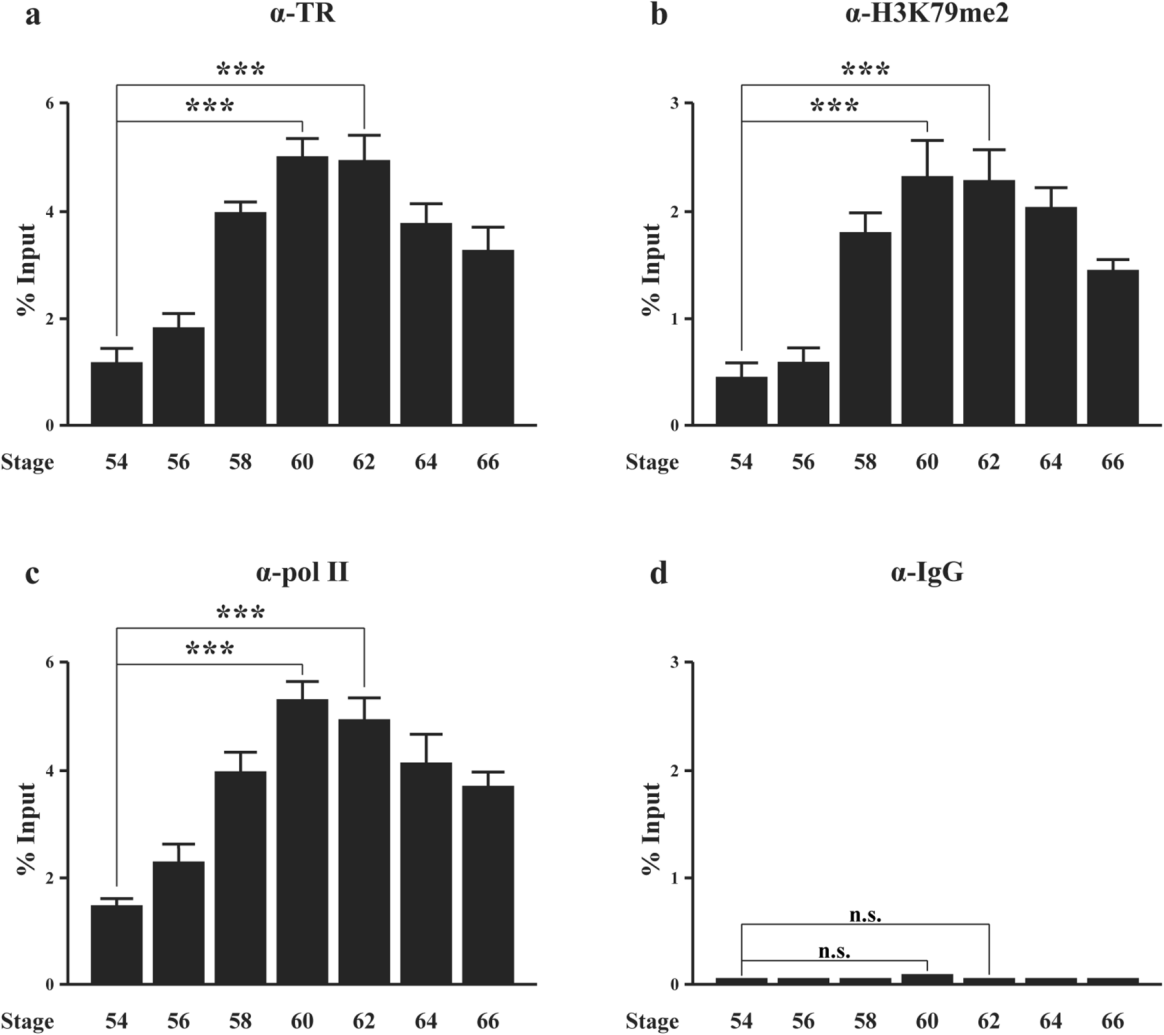
Peak levels of TR binding at the climax of intestinal metamorphosis correlates with peak levels of RNA polymerase II recruitment and H3K79 methylation in the intestine during natural metamorphosis. ChIP assays were performed with indicated antibodies on the intestine of tadpoles at different metamorphic stages. **a** TR binding to the TRE region of Mettl1 gene at metamorphic climax stages (stage 60 and 62) is increased compared to that at premetamorphic stage 54. After climax, TR binding is reduced in TRE region of Mettl1 gene. **b** The level of dimethylated H3K79 in the TRE region of Mettl1 gene peaks in the intestine at metamorphic climax. **c** RNA polymerase II recruitment to the TRE region of Mettl1 promoter also peaks at metamorphic climax period. **d** Only background ChIP signal is detected with the negative control IgG ChIP throughout metamorphosis. *n.s.* not significant

During metamorphosis, the plasma T3 level gradually increases from premetamorphic stage to peak at the metamorphic climax, and then decreases gradually [62]. To investigate TR binding to the Mettl1 TRE during natural metamorphosis, we performed ChIP assay on intestinal chromatin at different stages with antibodies against TR, RNA polymerase II, di-methylated H3K79 and normal IgG as a negative control. We found that TR occupancy at Mettl1 TRE gradually increased from premetamorphic stage to peak at the metamorphic climax, and then decreased toward the end of metamorphosis (Fig. 5a), mimicking the T3 level and consistent with the findings during T3-induced metamorphosis above. The recruitment of RNA polymerase II to Mettl1 TRE also peaked at metamorphic climax and so was the H3K79 methylation level (Fig. 5b, c), while only background signal was detected with the IgG control at all stages (Fig. 5d). These data suggest that increased TR binding to Mettl1 TRE leads to enhanced histone H3 K79 methylation and RNA polymerase II recruitment, consequently leading to the activation of Mettl1 expression during intestinal metamorphosis.

## Discussion/conclusion

Anuran metamorphosis is an important developmental process for studying the molecular mechanism of vertebrate postembryonic development. Mammalian embryos develop in the uterus and are under the maternal influence, while anurans develop externally and are independent of maternal effects. This benefit allows easy access and manipulation of anuran tadpoles during metamorphosis. In the last few decades, it has been revealed that TR plays an essential role in mediating the effects of T3 during metamorphosis [9–35, 63]. TR recruits cofactor complexes to the TRE regions of its target genes and activate or represses their expression in a T3-dependent manner. Therefore, to understand the molecular mechanisms underlying T3-regulation of anuran metamorphosis, it is critical to identify and characterize T3 target genes during metamorphosis. Through our earlier ChIP-on-chip analysis, Mettl1, encoding a tRNA methyltransferase, was found as a putative direct target of TR during intestinal metamorphosis. Our studies here demonstrate that Mettl1 is activated by TR directly via a TRE located upstream of the transcription start site during intestinal metamorphosis and its expression peaks at the climax of metamorphosis, implicating a critical role of Mettl1 during intestinal remodeling.

Consistent with the identification of Mettl1 by our earlier ChIP-on-chip assay, we discovered a putative TRE highly similar to the consensus TRE through bioinformatic analysis. The putative TRE is located at -1128 bp from the putative transcription start site of Mettl1, in agreement with the ChIP-on-chip data [40]. Our transcriptional studies on the promoter in the frog oocyte system demonstrated that the Mettl1 promoter can be activated by overexpressed TR/RXR in the presence of T3 and that this activation is dependent on the putative TRE, as its mutation abolished the activation. More importantly, our ChIP assay analyses of tadpole intestine during both natural and T3-induced metamorphosis revealed that TR is bound to the TRE region in the intestine and T3 induces the recruitment of RNA polymerase II and H3K79 methylation, a known activation histone mark catalyzed by a TR-coactivator methyltransferase Dot1L [61], during both natural and T3-induced metamorphosis. Furthermore, TR binding, histone H3K79 methylation, and RNA polymerase II recruitment all peak at the climax of metamorphosis when drastic intestinal remodeling, particularly larval epithelial cell death and adult epithelial stem cell formation/proliferation take place. These data suggest that the endogenous TRs bind to Mettl1 TRE region to regulate local histone modifications and recruit RNA polymerase II in the presence of T3, leading to the activation of Mettl1 expression to facilitate intestinal remodeling.

Consistent with the above, we observed that Mettl1 mRNA level is also induced by T3 during nature or T3-induced metamorphosis and correlates with the levels of H3K79 methylation and RNA polymerase II recruitment. In particular, peak levels of Mettl1 mRNA are present at the climax of metamorphosis, suggesting that Mettl1 is likely involved in epithelial transformation that takes place during this period.

Mettl1 contains a conserved methyltransf_4 domain that is essential to methylate the guanosine (m7G) of tRNA [41–43]. The methylation of m7G tRNA can regulate tRNA folding, stability, and/or function under certain growth conditions [47]. In mouse, the depletion of Mettl1 in embryonic stem cells (mESCs) reduces the expression of stem cell markers Nanog and KLF4 [47]. Furthermore, Mettl1 knockout reduces cell cycle progression and impairs self-renewal [47]. In addition, in mESCs, knockout of Wdr4, the cofactor of Mettl1, abolishes m7G tRNA modification and impairs mESCs proliferation and differentiation [47]. These observations suggest that Mettl1 is important for cell proliferation and/or stem cell function by regulating tRNA stability and/or function. Given the de novo development of the adult intestinal epithelial stem cells and their subsequent rapid proliferation at the climax of metamorphosis, it is templating to speculate that T3-induction of Mettl1 directly at the transcriptional level in the epithelium is critical for the formation and/or proliferation of adult intestinal epithelial stem cells during metamorphosis, which undoubtedly involves extensive regulation of the translation of many diverse proteins. It would be of interest in the future to test this by adopting gene editing approaches [64, 65] to knock out Mettl1 and study its effect on *Xenopus tropicalis* metamorphosis.

## Materials and methods

### Experimental animals

*Xenopus tropicalis* and *Xenopus laevis* were purchased from Nasco (For Atkinson, MI). Animal stages were determined in accordance with [66]. Premetamorphic tadpoles at stage 54 were treated with 10 nM T3, close to the physiological levels during metamorphosis [62], in the rearing water for 2 days. All animal care and treatments were performed as approved by the Animal Use and Care Committee of Eunice Kennedy Shriver National Institute of Child Health and Human Development of the National Institutes of Health.

### Quantitative RT-PCR (qRT-PCR)

Total RNA was isolated from tadpole intestine at indicated stages with Trizol^®^ reagent (Thermo Fisher Scientific, Waltham, MA, USA). The RNA concentration was measured by using a NanoDrop (Thermo Scientific). cDNA was synthesized from 2 μg of total RNA by using the High Capacity cDNA Archive kit (Applied Biosystems, Foster city, CA) in a 20 μl reaction. qRT-PCR was carried out by using SYBR Green PCR Master Mix on a StepOne Plus Real-Time PCR system (Applied Biosystems, Foster city, CA). The primers for qRT-PCR were used 5′-GGAACAGCATCAGGTGGAGT-3′ (forward), 5′-AATCCGAAACCTTCACTCGGA-3′ (reverse) for Mettl1, and 5′-CTATCCCCGCCAAACATCT-3′ (forward), 5′-CCATCTCAGCAGCTTCCTTC-3′ (reverse) for EF1α as an internal control.

### Bioinformatic identification of a putative TRE

The sequence of *Xenopus tropicalis* Mettl1 gene was obtained from ENSEMBL website (https://useast.ensembl.org/index.html) and the bioinformatical analysis tool NHR-Scan (http://www.cisreg.ca/cgi-bin/NHR-scan/nhr_scan.cgi) was used to search for putative TREs.

### Generation of promoter reporter constructs

The putative TRE is located at − 1128 bp from the predicted 5′-end of *Xenopus tropicalis* Mettl1. A 2815 bp length promoter region was PCR-amplified from genomic DNA with the primer pair: 5′-A GGGGTACCATCTAATATTGTGGTGCAGGGGTT-3′ (bearing KpnI site at its 5′-end) and 5′-CCGCTCGAGCGTCAGAGACATGCGACAAACATG-3′ (bearing XhoI site at its 5′-end). The PCR product was cloned into pGL4.10 firefly luciferase reporter vector (Promega, Madison, WI). Mutation of TRE (mTRE) was done by PCR with primers 5′-CAGAGAATCTAATTTTAACTGATTTTTTCAAACCTC-3′ and 5′-GAGGTTTGAAAAAATCAGTTAAAATTAGATTCTCTG-3′.

### Transcription assay in *Xenopus laevis* oocytes

Oocyte transcription assay was performed as described [59]. Briefly, the plasmids containing *Xenopus tropicalis* TRα and RXRβ [59] or GFP [55] were linearized and transcribed in vitro by using the mMESSAGE mMACHINE T7 transcription kit (Ambion, Grand Island, NY). The GFP mRNA or TR/RXR mRNA mixture (46 pg/oocyte) was injected into the cytoplasm of stage VI *Xenopus laevis* oocytes. Two hours later, the wild type or mutant TRE promoter luciferase reporter construct (115 pg/oocyte) was injected into the nuclei of these oocytes along with the internal control *Renilla* luciferase reporter phRG-TK (11.5 pg/oocyte). After incubation at 18 °C overnight in the presence or absence of 100 nM T3, groups of oocytes were harvested for dual luciferase assay by using the Dual-Luciferase-Reporter Assay kit (Promega, Madison, WI). The relative expression of firefly luciferase to *Renilla* luciferase was determined. The data shown here were representative of a few independent experiments with similar results.

### Chromatin immunoprecipitation (ChIP) assay

ChIP assay on *Xenopus tropicalis* tadpole intestines was performed as described previously with an antibody against TR (anti-TR) [59], RNA Polymerase II (abcam, Cambridge, MA), di-methylated histone H3K79 (abcam, Cambridge, MA) [60]. Normal IgG was used as a negative control. Each sample included 5 tadpole intestines. The immunoprecipitated DNA was analyzed by qPCR with SYBR Green PCR master Mix on a StepOne machine (Applied Biosystems, Foster City, CA). For analysis of Mettl1 TRE, the following primers were used: 5′-GAAATGTACAGCGTCCACCA-3′ and 5′-GGTGGTTACCAAACTGAGGGT-3′.

### Statistical analysis

All quantitative data are presented as mean ± S.E.M. (standard error of the mean) for three independent experiments. The differences between two groups were evaluated by a paired t-test. Significance values were *** *P *≤ 0.005.

## Data Availability

Not applicable.
